# ﻿First data on the Hirudinea fauna of lotic ecosystems of the Khanty-Mansi Autonomous Area (Russia)

**DOI:** 10.3897/zookeys.1082.71859

**Published:** 2022-01-19

**Authors:** Lyudmila I. Fedorova, Irina A. Kaygorodova

**Affiliations:** 1 Surgut State University, 1 Lenin Ave., Surgut 628412, Russia Surgut State University Surgut Russia; 2 Limnological Institute, Siberian Branch of Russian Academy of Sciences, 3 Ulan-Batorskaya St., Irkutsk 664033, Russia Limnological Institute, Siberian Branch of Russian Academy of Sciences Irkutsk Russia

**Keywords:** Hirudinea, new records, north of Western Siberia rivers, species distribution, species diversity

## Abstract

Hirudinea, a small and ecologically important group of aquatic organisms, is poorly studied in northern Eurasia. In this study, we demyth the idea of the faunistic poverty of this region and present the first findings of rheophilic leeches from the Khanty-Mansi Autonomous Area, Russia. Investigation of 25 rivers (Severnaya Sosva, Ob, Konda-Irtysh, and Bolshoi Yugan river basins) resulted in finding 10 leech species with parasitic and non-parasitic life strategies. These species belong to two orders (Rhynchobdellida and Arhynchobdellida), three families (Glossiphoniidae, Piscicolidae, and Erpobdellidae) and six genera (*Alboglossiphonia*, *Glossiphonia*, *Helobdella*, *Hemiclepsis*, *Piscicola*, and *Erpobdella*). Five species, *A.hyalina*, *G.verrucata*, *E.monostriata*, *E.vilnensis*, and potentially new morphological species of piscine leeches *Piscicola* sp., have been discovered for the first time in Western Siberia. Data on species diversity of rheophilic leeches include the exact systematic position for all leech taxa. Each species from the list is supplemented with information about its geographical distribution.

## ﻿Introduction

Khanty-Mansi Autonomous Area is located in the central part of the West Siberian Plain, stretching for almost 1400 km from the Ural ridge in the west to the Ob-Yenisei watershed in the east, and extending about 800 km from north to south (https://www.geografia.ru). The region has an extensive system of watercourses of various types, of which the Ob and Irtysh rivers are among the largest in Russia. The total length of the hydraulic network is about 100,000 km (Dobrinskii and Plotnikov 1997). The taiga rivers in the region are characterized by wide floodplains and valleys, small slopes, and low flow rates (Frolov and Sazonov 2004). The extended spring-summer flood, freshets in the warm season, and backwater phenomena contribute to a strong watering of watersheds with the formation of lars (floodplain swamps) and sors (seasonal lakes formed in flooded low-lying areas).

Many rivers of the Khanty-Mansi Autonomous Area are undergoing anthropogenic transformations mainly associated with large-scale oil production. Greater damage to ecosystems is caused not only by oil pollution *per se* (Picunov and Bortnikova 2005; Uslamin et al. 2019) but also by salinization of aquatic ecosystems due to the outflow of sodium chloride water to the surface during oil recovery (Moskovchenko et al. 2017). Local emergencies at the sites of production, processing, and transportation of hydrocarbon, often raw, lead to ingress of an oily liquid into the catchments of small watercourses with its subsequent migration to larger water systems (Zakharov et al. 2011). At the same time, the reduced ability of northern rivers for biological self-purification aggravates the vulnerability of aquatic biocenoses (Yakovlev 2005).

Leeches are an integral component of any aquatic biocenoses. Their role is especially significant in freshwater benthic communities of coastal zones where they are the most abundant (Adamiak-Brud et al. 2016). Ecosystem relationships between leeches and other organisms are highly diverse. Non-parasitic representatives of this group are a source of nutrient-valuable substances, and are, therefore, attractive to predatory mammals, semi-aquatic birds, fishes, and amphibians (Lukin 1976). In addition to their role in the food web, leeches are of interest as accumulators of toxicants (Lapkina and Flerov 1980; Romanova and Klimina 2009) and as bioindicators of water pollution (Bezmaternykh 2007; Martins et al. 2008; Fedorova 2020). The ecological role of parasitic forms is not limited to regulating the number of host species by weakening and creating conditions for the development of infections. Leeches are directly related to the transmission of bacterial and viral infections (Ahne 1985; Mulcahy et al. 1990; Faisal and Schulz 2009), as well as hematozoa, including trematodes, cestodes, nematodes (Demshin 1975), and parasitic flagellates (Khan 1976; Khamnueva and Pronin 2004; Burreson 2007), which are considered to be pathogenic organisms for aquatic animals.

To date, no object-orientated studies on the leech fauna of the Khanty-Mansi Area have been performed. The only study reports on three species of Hirudinea from the Khanty-Mansi lakes (Uslamin et al. 2019): the widespread Palaearctic leech, *Helobdellastagnalis* (Linnaeus, 1758), the medicinal leech, *Hirudomedicinalis* (Linnaeus, 1758), which is unexpected in this region, and the easily recognizable stagnophilic *Erpobdellanigricollis* (Brandes, 1900). The lack of information on the hirudofauna and only a few studies on other groups of rheophilic hydrofauna (Stepanova 2008; Semyonova and Aleksyuk 2010; Sharapova and Babushkin 2013) can be explained by the difficulties in accessing lotic ecosystems due to the peculiarities of their hydrological regime in this region.

This paper presents the first purposeful study of the leech fauna from the watercourses in the Khanty-Mansi Autonomous Area, debunking the myth the aquatic invertebrate fauna in the north of Western Siberia is impoverished.

## ﻿Materials and methods

**Figure 1.**
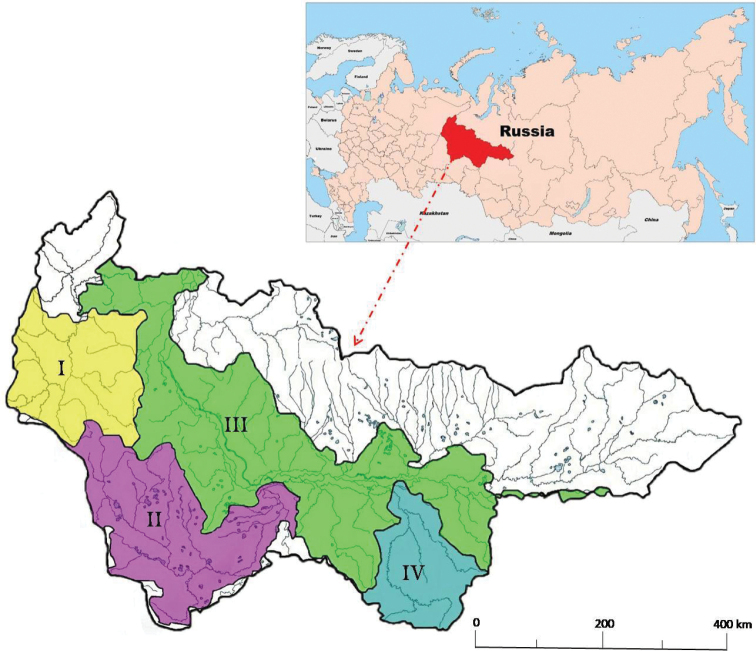
Schematic map of geographic location of the Khanty-Mansi Autonomous Area and studied lotic systems. River basins: I = Severnaya Sosva, II = Konda-Irtysh, III = Ob, and IV = Bolshoi Yugan.

Leech sampling was carried out from 6 June to 20 September 2020 at 44 locations along 25 large and small watercourses belonging to the Bolshoi Yugan, Severnaya Sosva, Konda-Irtysh, and Ob watershed basins (Fig. 1).

The use of conventional hydrobiological equipment (sweep net, dredge, scraper, bottom grab, etc.) is less effective in catching leeches than for many other aquatic invertebrates; therefore, the collection of leeches was done manually. To do this, we examined aquatic plants and potential host animals to detect parasitic and predatory leeches, as well as various underwater objects (rotten tree, driftwood, stones, etc.) to which leeches can attach. Collected individuals were fixed after preliminary anesthesia in a low-concentration alcohol solution and kept in 80% ethanol. Morphological analysis was conducted using a stereomicroscope MSP-2 var. 2 (LOMO). Species affiliation was determined using existing systematic keys (Lukin 1976; Nesemann and Neubert 1999). The external morphology of identified leeches was in agreement with the relevant species description. All taxonomic names were given according to the current classification of the group. The collection of leech species with voucher specimens was deposited at Surgut State University, Russia.

## ﻿Results

An object-oriented hydrobiological survey carried out in the warm season of 2020 resulted in finding leeches in 20 of 25 examined watercourses of the Khanty-Mansi Autonomous Area. This indicates a high frequency of their occurrence in nature. Leeches inhabit at least 88% of the region’s rivers. However, not all surveyed water bodies turned out to be suitable for leeches. In particular, we could not find them in some watercourses, namely, in the Shaitanka rivers, Bezymyannyi Creek (Severnaya Sosva river basin), in two nameless brooks (Ob river basin), and the Pach-peu River (Bolshoi Yugan river basin). Very cold water, fast current, and, hence, biotic poverty of streams, creeks, and brooks make these habitats less suitable for leeches. There were no leeches in the navigable sections of the Irtysh. In the Pach-peu River, leeches were absent probably due to poor water quality.

In this first faunistic leech species list, 10 species were documented. The species diversity includes leeches from two orders (Rhynchobdellida and Arhynchobdellida), three families (Glossiphoniidae, Piscicolidae, and Erpobdellidae), and six genera (*Alboglossiphonia*, *Helobdella*, *Hemiclepsis*, *Glossiphonia*, *Piscicola*, and *Erpobdella*). Species composition includes both free-living and parasitic freshwater leeches. Parasitic leeches form the majority of the region’s hirudofauna and are represented by seven species, including representatives of five genera: *Alboglossiphoniahyalina* (Müller, 1774), *Helobdellastagnalis* (Linnaeus, 1758), *Hemiclepsismarginata* (Müller, 1774), *Glossiphoniacomplanata* (Linnaeus, 1758), *Glossiphoniaconcolor* (Aphathy, 1888), *Glossiphoniaverrucata* (F. Müller, 1844), and *Piscicola* sp. Among free-living macrophagous leeches, there were only three *Erpobdella* species: *E.monostriata* (Lindenfeld & Pietruszynski, 1890), *E.octoculata* (Linnaeus, 1758), and *E.vilnensis* (Liskiewicz, 1925).

Our study did not confirm the information provided in the literature about findings of *Hirudomedicinalis* Linnaeus, 1758 and *Erpobdellanigricollis* (Brandes, 1900) (Uslamin et al. 2019) within the Khanty-Mansi Autonomous Area. According to Nesemann and Neubert (1999), *E.nigricollis* belongs to the potamic fauna, with a preference for large rivers; in contrast, Lukin (1976) ranked this species as being typical of small lakes and naturally stagnant water bodies located in the floodplains of rivers. Most Russian researchers (e.g., Baturina et al. 2020) tend to agree with Lukin’s opinion. If the presence/absence of *E.nigricollis* in the watercourses of the Khanty-Mansi Area is discussable, the presence of *H.medicinalis* in the north of Western Siberia is much more questionable and needs additional verification. The range of this medicinal leech species corresponds to areas initially covered by deciduous tree forests and does not extend beyond Central and Northern Europe (Utevsky et al. 2010).

The checklist includes both widespread Palaearctic species (*G.complanata*, *H.marginata*, and *E.octoculata*) and widespread Holarctic species (*H.stagnalis*). Five species, *A.hyalina*, *G.verrucata*, *E.monostriata*, *E.vilnensis*, and *Piscicola* sp. were discovered for the first time in Western Siberia. In this paper, a single specimen of *Piscicola* sp. is cautiously referred to as an unidentified species because its morphology differs from all currently described species. It is highly probable that this unidentified species is potentially new to science. Clarification of its attribution and its description will require additional biological material and in-depth analysis.

The species composition of the Khanty-Mansi hirudofauna has an uneven distribution (Table 1). The greatest species diversity was observed in the Ob basin: nine of 10 species from the regional list (except *A.hyalina*) inhabit its watercourses. This is probably due to the flat nature of the ramified water network, numerous tributaries carrying nutrients from a vast territory, and a high level of self-purification of rivers.

**Table 1.** Species composition of the Hirudinea fauna in lotic ecosystems of the Khanty-Mansi Area, with an estimate of occurrence frequency (rather rare +, common ++, everywhere +++).

Taxa	River basins			
	Ob	Konda-Irtysh	Severnaya Sosva	Bolshoi Yugan
*Alboglossiphoniahyalina*	–	+	–	–
*Helobdellastagnalis*	++	+++	+	++
*Hemiclepsismarginata*	++	++	–	+
*Glossiphoniacomplanata*	+	++	–	+
*Glossiphoniaconcolor*	++	+	+	+
*Glossiphoniaverrucata*	+	–	–	–
*Piscicola* sp.	+	–	–	–
*Erpobdellaoctoculata*	++	++	++	+
*Erpobdellamonostriata*	++	+	+	+
*Erpobdellavilnensis*	+	–	–	+

Within the Severnaya Sosva river network, the Hirudinea fauna was the least diverse (Table 1). Due to natural inaccessibility, sampling in this area was carried out less intensively than in other examined basins. However, the four species found here represented every possible variety of life strategies of leeches: the parasitic *G.concolor*, the small predator *H.stagnalis*, and the free-living macrophagous *E.octoculata* and *E.monostriata*. These leeches were found in the Yatria and Schekurya rivers.

Leeches from the Konda-Irtysh and Bolshoi Yugan watershed basins were represented by seven species: *H.marginata*, *G.complanata*, *G.concolor*, *H.stagnalis*, *E.octoculata*, *E.monostriata*, and *A.hyalina* or *E.vilnensis* depending on the basin (Table 1). Among watercourses of the Konda-Irtysh system, the highest diversity was observed in the main riverbed of the Irtysh. The leech populations of the Bolshoi Yugan basin were sparse, and individuals were not numerous.

Species *H.stagnalis*, *G.concolor*, *E.octoculata*, and *E.monostriata* are widespread in the rivers of the Khanty-Mansi Area, whereas *G.verrucata* and *E.vilnensis* are rare for the eastern Palaearctic obviously prefer the southern areas of the region (Table 1).

Listed below is the information about the species composition of Hirudinea fauna of the the Khanty-Mansi Autonomous Area, with systematic position, geographical distribution, and habitat coordinates for each species.

## ﻿Systematics

**Phylum Annelida Lamarck, 1809**

**Class Clitellata Michaelsen, 1919**

**Subclass Hirudinea Lamarck, 1818 (synonym Hirudinida)**

**Order Rhynchobdellida Blanchard, 1894**

### ﻿Family Glossiphoniidae Vaillant, 1890

#### Genus *Alboglossiphonia* Lukin, 1976

##### ﻿ Alboglossiphonia hyalina

Taxon classificationAnimaliaRhynchobdellidaGlossiphoniidae

(Müller, 1774)

35F98366-FAFA-5AA5-AB1D-32E2A63346FB

Hirudo hyalina Müller 1774
Clepsine hyalina Moqun-Tandon 1826
Glossobdella hyalina De Blainville1827
Glossiphonia heteroclita f. hyalina Pawlowski 1936

###### Geographical distribution.

Palaearctic region. This species is rare in Europe (Nesemann and Neubert 1999) but abundant in Eastern Siberia (Kaygorodova et al. 2013).

###### Location.

Not an abundant species. Point occurrence in floodplain water bodies of the Irtysh River (61°0'58"N, 69°9'26"E).

#### Genus *Glossiphonia* Johnson, 1816

##### ﻿ Glossiphonia complanata

Taxon classificationAnimaliaRhynchobdellidaGlossiphoniidae

(Linnaeus, 1758)

D530E904-7C8A-5243-B6B7-5708BEEE05AF

Hirudo complanata Linnaeus, 1758
Glossiphonia tuberculate Johnson, 1816
Glossiphonia complanata Blanchard, 1894

###### Geographical distribution.

Palaearctic region. Previously mentioned as Holarctic. However, recent molecular studies confuted its findings in North America (Williams et al. 2013; Kaygorodova et al. 2020).

###### Locations.

Ob River (61°5'38"N, 69°27'38.6"E; 60°57'56"N, 68°46'38"E), Kabaniy stream (60°54'4"N, 68°42'55"E), Okhotnichiy stream (60°56'9"N, 68°41'52"E), Irtysh River (61°0'58"N, 69°9'26"E; 61°0'14"N, 68°59'10"E; 60°59'57"N, 68°59'19"E; 60°59'37"N, 68°59'22"E), Wachem-peu River (60°16'34.0"N, 73°55'18.0"E), Lungunigyi River (60°11'43.0"N, 74°12'20.0"E), Ugutka River (60°29'26.0"N, 74°03'45.0"E).

##### ﻿ Glossiphonia concolor

Taxon classificationAnimaliaRhynchobdellidaGlossiphoniidae

(Aphathy, 1888)

0D00C530-2E8D-5045-93D9-AEAFB82343D4

Clepsine concolor Apathy, 1888
Glossiphonia concolor Livanow, 1903

###### Geographical distribution.

Palaearctic region. Distributed in northern, central, and eastern Europe (Nesemann and Neubert 1999). There is information about its occurrence in Iran (Darabi-Darestani et al. 2016) and occasionally in Eastern Siberia (Kaygorodova and Pronin 2013).

###### Locations.

Schekurya River (64°15'52.35"N, 60°54'23.98"E), Yatria River (64°15'50"N, 60°52'39"E), Ob River (61°5'38.0"N, 69°27'38.6"E; 61°5'47"N, 69°27'46"E; 60°57'13"N, 68°39'27"E), Saima River (61°14'41.1"N, 73°25'09.5"E; 61°14'32.6"N, 73°24'53.1"E), Shaitanskaya River (61°4'50.4"N, 69°28'51.9"E), Zhivoy stream (60°53'22"N, 68°41'40"E), Irtysh River (61°1'21"N, 69°8'20"E; 61°0'58"N, 69°9'26"E), Bolshoi Yugan River (60°17'38.0"N, 73°53'32.0"E), Malyi Yugan River (60°31'22.3"N, 74°28'01.9"E), and Wachem-peu River (60°16'17.0"N, 73°55'22.1"E).

##### ﻿ Glossiphonia verrucata

Taxon classificationAnimaliaRhynchobdellidaGlossiphoniidae

(F. Müller, 1844)

EC20FEA3-FDC7-5F93-B5F9-622F93D15B35

Clepsine verrucata Müller, 1844
Glossiphonia verrucata Johansson, 1909
Batracobdella verrucata Pawlowski, 1936
Boreobdella verrucata Lukin, 1956

###### Geographical distribution.

Palaearctic region. Although *G.verrucata* is a rare species, it nevertheless has an extensive distribution within the Palaearctic. The boreal species inhabits northern Eurasia (Lukin 1976). There are recent findings from the Kharbey lake system, Russian North (Baturina et al. 2020) and the basins of the Lena River and Lake Baikal, Eastern Siberia (Kaygorodova et al. 2020).

###### Locations.

Not an abundant species. Point occurrence in Shaitanskaya River (61°4'50.4"N, 69°28'51.9"E) and Kabaniy stream (60°54'4"N, 68°42'55"E).

#### Genus *Helobdella* Blanchard, 1896

##### ﻿ Helobdella stagnalis

Taxon classificationAnimaliaRhynchobdellidaGlossiphoniidae

(Linnaeus, 1758)

745EBD01-D5A2-5552-AF81-F03C8F276D64

Hirudo stagnalis Linnaeus, 1758
Glossiphonia stagnalis Blanchard, 1894
Glossiphonia (Helobdella) stagnalis Moore, 1922
Bakedebdella gibbosa Sciacchitiano, 1939

###### Geographical distribution.

Transpalaearctic species. This is one of the most common leech species inhabiting freshwater ecosystems in Eurasia.

###### Locations.

Yatria River (64°15'50"N, 60°52'39"E), Ob River (61°5'38.0"N, 69°27'38.6"E; 60°57'13"N, 68°39'27"E), Saima River (61°14'41.1"N, 73°25'09.5"E), Shaitanskaya River (61°4'50.4"N, 69°28'51.9"E), Zhivoy stream (60°53'22"N, 68°41'40"E), Kabaniy stream (60°54'4"N, 68°42'55"E), Okhotnichiy stream (60°56'9"N, 68°41'52"E), Sredniy stream (60°55'24"N, 68°42'46"E), Irtysh River (61°1'21"N, 69°8'20"E; 61°0'58"N, 69°9'26"E; 61°0'14"N, 68°59'10"E; 60°59'57"N, 68°59'19"E; 60°59'37"N, 68°59'22"E), Mamontovyi creek (60°57'18"N, 68°32'27"E), Malyi Yugan River (60°31'22.3"N, 74°28'01.9"E), Bolshoi Yugan River (60°17'38.0"N, 73°53'32.0"E; 60°17'33.0"N, 73°53'38.0"E), Lungunigyi River (60°11'43.0"N, 74°12'20.0"E), Ugutka River (60°29'17.0"N, 74°03'41.0"E).

#### Genus *Hemiclepsis* Vejdovsky, 1884

##### ﻿ Hemiclepsis marginata

Taxon classificationAnimaliaRhynchobdellidaGlossiphoniidae

(Müller, 1774)

3DC0E780-0029-535D-9165-87411987BB3E

Hirudo marginata O. F. Müller, 1774
Piscicola marginata Moquin-Tandon, 1827
Clepsine marginata F. Müller, 1844
Hemiclepsis marginata Harding, 1910

###### Geographical distribution.

Palaearctic region. Species has wide but uneven distribution. In Europe, this species is common in countries with temperate climates. Rarely found in North Africa. It has a nonuniform distribution in the Caucasus, Central Asia, Western and Eastern Siberia, the Far East, China, and Japan (Lukin 1976).

###### Locations.

Ob River (61°5'38.04"N, 69°27'38.66"E; 60°57'56"N, 68°46'38"E), Shaitanskaya River (61°4'50.4"N, 69°28'51.9"E), Zhivoy stream (60°53'22"N, 68°41'40"E), Mukhrinka River (60°53'42"N, 68°42'51"E), Kabaniy stream (60°54'4"N, 68°42'55"E), Okhotnichiy stream (60°56'9"N, 68°41'52"E), Sredniy stream (60°55'24"N, 68°42'46"E), Irtysh River (61°0'58"N, 69°9'26"E; 61°0'14"N, 68°59'10"E; 60°59'57"N, 68°59'19"E; 60°59'37"N, 68°59'22"E), Mamontovyi creek (60°57'18"N, 68°32'27"E), Bolshoi Yugan River (60°17'33.0"N, 73°53'38.0"E).

### ﻿Family Piscicolidae Johnston, 1865 (synonym Ichthyiobdellidae Leuckart, 1863)

#### Genus *Piscicola* Blanville, 1818

##### ﻿ Piscicola

Taxon classificationAnimaliaRhynchobdellidaPiscicolidae

sp.

15E8BA98-C871-5A0C-9F94-96DDF782B662

###### New species records.

A single specimen from the Chumpas River (61°16'24"N, 74°42'32"E).

###### Morphological characteristics.

Piscine leech has middle size, its body length is 22 mm and diameter is 3.5 mm. Sucker size is commensurate with the width of the body. Dorsal pigmentation is absent, unlike the widespread *P.geometra* or other known species.

### ﻿Family Erpobdellidae Blanchard, 1894

#### Genus *Erpobdella* Blainville, 1918

##### ﻿ Erpobdella octoculata

Taxon classificationAnimaliaRhynchobdellidaErpobdellidae

(Linnaeus, 1758)

B9ECE143-4A8F-578B-B1A5-911EB4C2204D

Hirudo octoculata Linnaeus, 1758
Herpobdella octoculata Johansson, 1910
Herpobdella octomaculata Pawlowski, 1935

###### Geographical distribution.

Widespread in the Palaearctic region.

###### Locations.

Yatria River (64°15'50"N, 60°52'39"E), Ob River (61° 5'38.0"N, 69°27'38.6"E; 60°57'13"N, 68°39'27"E), Saima River (61°14'32.6"N, 73°24'53.1"E), Shaitanskaya River (61°4'50.4"N, 69°28'51.9"E), Mukhrinka River (60°53'42"N, 68°42'51"E), Zhivoy stream (60°53'22"N, 68°41'40"E), Kabaniy stream (60°54'4"N, 68°42'55"E), Okhotnichiy stream (60°56'9"N, 68°41'52"E), Sredniy stream (60°55'24"N, 68°42'46"E), Irtysh River (61°1'21"N, 69°8'20"E; 61°0'58"N, 69°9'26"E), Mamontovyi creek (60°57'18"N, 68°32'27"E), Malyi Yugan River (60°31'22.3"N, 74°28'01.9"E), Bolshoi Yugan River (60°17'38.0"N, 73°53'32.0"E), Negus-yah River (60°11'55.0"N, 74°12'54.0"E; 60°11'19.0"N, 74°14'34.0"E), Ugutka River (60°29'17.0"N, 74°03'41.0"E; 60°29'26.0"N, 74°03'45.0"E).

##### ﻿Species: Erpobdella monostriata

Taxon classificationAnimaliaRhynchobdellidaErpobdellidae

(Lindenfeld et Pietruszynski, 1890)

802411C0-EA31-5573-AD4A-AEB481BE24D1

Nephelis octoculata var. monostriata Lindenfeld & Pietruszynski, 1890
Erpobdella vilnensis (Liskiewitz, 1925) in part

###### Geographical distribution.

Palaearctic region. This species occurs in Europe from the Netherlands (Haaren et al. 2004) in the west to the Voronezh region of Russia in the east (Utevsky et al. 2015). It has been recently reported in East Kazakhstan (Kaygorodova & Fedorova, 2016). This study reports the first finding in the north of Western Siberia.

###### Locations.

Schekurya River (64°15'52.35"N, 60°54'23.98"E), Ob River (61°15'27.4"N, 73°20'56.9"E), Saima River (61°14'41.1"N, 73°25'09.5"E), Shaitanskaya River (61°4'50.4"N, 69°28'51.9"E), Mukhrinka River (60°53'42"N, 68°42'51"E), Zhivoy stream (60°53'22"N, 68°41'40"E), Kabaniy stream (60°54'4"N, 68°42'55"E), Okhotnichiy stream (60°56'9"N, 68°41'52"E), Sredniy stream (60°55'24"N, 68°42'46"E), Irtysh River (61°1'21"N, 69°8'20"E; 61°0'58"N, 69°9'26"E), Bolshoi Yugan River (60°17'33.0"N, 73°53'38.0"E), Wachem-peu River (60°16'17.0"N, 73°55'22.1"E).

##### ﻿ Erpobdella vilnensis

Taxon classificationAnimaliaRhynchobdellidaErpobdellidae

(Liskiewicz, 1925)

DBDA8206-F39A-5A47-8611-21C777E602E4

Nephelis testacea f. nigricollis Brandes, 1900
Herpobdella testacea var. nigricollis Johansson, 1929

###### Geographical distribution.

Palaearctic region. *Erpobdellavilnensis* is rather a common leech species that occurs in Central, Eastern, and Southeastern Europe (Nesemann and Neubert 1999). The easternmost distribution records were from Kyrgyzstan (Jueg et al. 2013) and eastern Kazakhstan (Kaygorodova and Fedorova 2016).

###### Locations.

Ob River (60°57'56"N, 68°46'38"E; 61°15'27.4"N, 73°20'56.9"E), Shaitanskaya River (61°4'50.4"N, 69°28'51.9"E), Negus-yah River (60°11'55.0"N, 74°12'54.0"E), Lungunigyi River (60°11'43.0"N, 74°12'20.0"E).

## Supplementary Material

XML Treatment for Alboglossiphonia hyalina

XML Treatment for Glossiphonia complanata

XML Treatment for Glossiphonia concolor

XML Treatment for Glossiphonia verrucata

XML Treatment for Helobdella stagnalis

XML Treatment for Hemiclepsis marginata

XML Treatment for Piscicola

XML Treatment for Erpobdella octoculata

XML Treatment for Erpobdella monostriata

XML Treatment for Erpobdella vilnensis

## References

[B1] Adamiak-Brud Ż Bielecki A Kobak J Jabłońska-Barna I (2016) Rate of short-term colonization and distribution of leeches (Clitellata: Hirudinia) on artificial substrates. Journal of Zoology 299: 191–201. 10.1111/jzo.12341

[B2] Ahne W (1985) *Argulusfoliaceus* L. and *Piscicolageometra* L. as mechanical vectors of spring viraemia of carp virus (SVCV). Journal of Fish Diseases 8: 241–242. 10.1111/j.1365-2761.1985.tb01220.x

[B3] Baturina MA Kaygorodova IA Loskutova OA (2020) New data on species diversity of Annelida (Oligochaeta, Huridinea) in the Kharbey lakes system, Bolchezemelskaya tundra (Russia). ZooKeys 910: 43–78. 10.3897/zookeys.910.48486 32099515 PMC7026200

[B4] Bezmaternykh DM (2007) Zoobenthos as an indicator of ecological state of aquatic ecosystems in Western Siberia: an analytical review. Ekologiya 85: 1–86.

[B5] Burreson EM (2007) Hemoflagellates of Oregon marine fishes with the description of new species of *Trypanosoma* and *Trypanoplasma*. Journal of Parasitology 93(6): 1442–1451. 10.1645/GE-1220.1 18314692

[B6] Darabi-Darestani K Sari A Sarafrazi A (2016) Five new records and annotated checklist of the leeches (Annelida: Hirudinida) of Iran. Zootaxa 4170(1): 041–070. 10.11646/zootaxa.4170.1.2 27701273

[B7] Demshin NI (1975) Oligochaeta and Hirudinea as Intermediate Hosts of Helminthes. Nauka, Novosibirsk, 190 pp.

[B8] Dobrinskii LN Plotnikov VV (1997) Ecology of the Khanty-Mansi Autonomous Okrug. Tyumen': SoftDizayn, 288 pp.

[B9] Faisal M Schulz CA (2009) Detection of viral hemorrhagic septicemia virus (VHSV) from the leech *Myzobdellalugubris* Leidy, 1851. Parasites & Vectors 282(1): e45. 10.1186/1756-3305-2-45 PMC2761889 19785752

[B10] Fedorova LI (2020) Environmental factors influence on leeches distribution in the middle reaches of the Irtysh River. Samara Journal of Science 9(4): 159–164. 10.17816/snv202094124

[B11] Frolov RD Sazonov AA (2004) Prospects of development internal waterways XMAO. Vestnik of Volga State University of Water Transport 8: 94–101.

[B12] Haaren T Hop H Soes M Tempelman D (2004) The freshwater leeches (Hirudinea) of the Netherlands. Lauterbornia 52: 113–131.

[B13] Jueg U Grosser C Pešić V (2013) Bemerkungen zur Egelfauna (Hirudinea) von Kirgistan. Lauterbornia 76: 103–109.

[B14] Kaygorodova IA Fedorova LI (2016) The first data on species diversity of leeches (Hirudinea) in the Irtysh River Basin, East Kazakhstan. Zootaxa 4144(2): 287–290. 10.11646/zootaxa.4144.2.10 27470855

[B15] Kaygorodova IA Pronin NM (2013) New records of Lake Baikal leech fauna: species diversity and spatial distribution in Chivyrkuy Gulf. The Scientific World Journal 2013: 1–10. 10.1155/2013/206590 PMC3690781 23844382

[B16] Kaygorodova IA Bolbat NB Bolbat AV (2020) Species delimitation through DNA barcoding of freshwater leeches of the *Glossiphonia* genus (Hirudinea: Glossiphoniidae) from Eastern Siberia, Russia. Journal of Zoological Systematics and Evolutionary Research 58: 1437–1446. 10.1111/jzs.12385

[B17] Kaygorodova IA Dzyuba EV Sorokovikova NV (2013) First records of potamic leech fauna of Eastern Siberia, Russia. Dataset Papers in Biology 2013: 1–6. 10.7167/2013/362683

[B18] Khamnueva TR Pronin NM (2004) New kinetoplastid species (Kinetoplastida: Kinetoplastidea). In: Timoshkin OA (Ed.) Index of Animal Species in Inhabiting Lake Baikal and its Area.1, Book 2. Nauka, Novosibirsk, 1255–1260.

[B19] Khan RA (1976) The life cycle of *Trypanosomamurmanensis* Nikitin. Canadian Journal of Zoology 54(11): 1840–1849. 10.1139/z76-214 991015

[B20] Lapkina LN Flerov BA (1980) Using leeches to identify pesticides in water. Hydrobiological Journal 3: 113–118.

[B21] Lukin EI (1976) The Leech Fauna of the Soviet Union: Leeches in Fresh and Brackish Water Bodies. Nauka, Leningrad, 284 pp.

[B22] Martins RT Stephan NNC Alves RG (2008) Tubificidae (Annelida: Oligochaeta) as an indicator of water quality in an urban stream in southeast Brazil. Acta Limnologica Brasiliensia 20(3): 221–226.

[B23] Moskovchenko DV Babushkin AG Ubaidulaev AA (2017) Salt pollution of surface water in oil fields of Khanty-Mansi Autonomous Area-Yugra. Water Resources 44(1): 91–102. 10.7868/S0321059617010102

[B24] Mulcahy D Klaybor D Batts WN (1990) Isolation of infectious hematopoietic necrosis virus from a leech (*Piscicolasalmositica*) and a copepod (*Salminocola* sp.), ectoparasites of sockeye salmon *Oncorhynchusnerka*. Diseases of Aquatic Organisms 8: 29–34. 10.3354/dao008029

[B25] Nesemann H Neubert E (1999) Clitellata, Branchiobdellada, Acanthobdellada, Hirudinea. Spectrum Akademischer Verlag, Berlin, 6(2): 1–178.

[B26] Picunov SV Bortnikova SB (2005) Dynamics of pollution of small rivers in oil productions regions (by the example of the Lyukkolekegan and Chernaya rivers, Nizhnevartovsky district, KHMAO). Geology. Geophysics and Development of Oil and Gas Fields 12: 27–33.

[B27] Romanova EM Klimina OM (2009) The role of leeches in the biological mechanism of accumulation of toxicants. Vestnik of Ulyanovsk State Agricultural Academy 1(9): 85–88.

[B28] Semyonova LA Aleksyuk VA (2010) Zooplankton of the Lower Ob’ River. Bulletin of Ecology, Forestry and Landscape Science 10: 156–169

[B29] Sharapova TA Babushkin ES (2013) Comparison of zoobenthos and zooperiphyton of large and medium rivers. Contemporary Problems of Ecology 6(6): 622–627. 10.1134/S1995425513060097

[B30] Stepanova VB (2008) Macrozoobenthos of the lower Ob’. Bulletin of Ecology, Forestry and Landscape Science 9: 155–162.

[B31] Uslamin DV Aleshina OA Gashev SN Gradova AV (2019) Characteristics of the species composition and structure of macrozoobenthos in taiga lakes in oil-producing regions in western Siberia. Inland Water Biology 12: 306–316. 10.1134/S1995082919030179

[B32] Utevsky S Dubov PG Prokin AA (2015) First Russian record of *Erpobdellamonostriata*: DNA barcoding and geographical distribution. Spitaxa 38(2): 161–168.

[B33] Utevsky S Zagmajster M Atemasov A Zinenko O Utevska O Utevsky A Trontelj P (2010) Distribution and status of medicinal leeches (genus *Hirudo*) in the Western Palaearctic: anthropogenic, ecological, or historical effects? Aquatic Conservation: Marine and Freshwater Ecosystems 20: 198–210. 10.1002/aqc.1071

[B34] Williams B Gelder S Proctor H Coltman D (2013) Molecular phylogeny of North American Branchiobdellida (Annelida: Clitellata). Molecular Phylogenetics and Evolution 66(1): 30–42. 10.1016/j.ympev.2012.09.002 22995849

[B35] Yakovlev VA (2005) Freshwater Zoobenthos of Northern Fennoscandia (Diversity, Structure and Anthropogenic Dynamics) Apatity. Part 1. Kola Scientific Center of the Russian Academy of Sciences, Syktyvkar, 161 pp.

[B36] Zakharov AB Loskutova OA Fefilova EB Khokhlova LG Shubin YP (2011) Communities of Hydrobionts in Oil-polluted Water Areas of the Pechora River Basin. Kola Scientific Center of the Russian Academy of Sciences, Syktyvkar, 268 pp.

